# Performance of a 3D convolutional neural network in the detection of hypoperfusion at CT pulmonary angiography in patients with chronic pulmonary embolism: a feasibility study

**DOI:** 10.1186/s41747-021-00235-z

**Published:** 2021-09-24

**Authors:** Tuomas Vainio, Teemu Mäkelä, Sauli Savolainen, Marko Kangasniemi

**Affiliations:** 1 HUS Medical Imaging Center, Radiology, University of Helsinki and Helsinki University Hospital, P.O. Box 340 (Haartmaninkatu 4), FI-00290 Helsinki, Finland; 2 Department of Physics, University of Helsinki, P.O. Box 64, FI-00014 Helsinki, Finland

**Keywords:** Computed tomography angiography, Deep learning, Feasibility Studies, Neural networks (computer), Pulmonary embolism

## Abstract

**Background:**

Chronic pulmonary embolism (CPE) is a life-threatening disease easily misdiagnosed on computed tomography. We investigated a three-dimensional convolutional neural network (CNN) algorithm for detecting hypoperfusion in CPE from computed tomography pulmonary angiography (CTPA).

**Methods:**

Preoperative CTPA of 25 patients with CPE and 25 without pulmonary embolism were selected. We applied a 48%–12%–40% training-validation-testing split (12 positive and 12 negative CTPA volumes for training, 3 positives and 3 negatives for validation, 10 positives and 10 negatives for testing). The median number of axial images per CTPA was 335 (min–max, 111–570). Expert manual segmentations were used as training and testing targets. The CNN output was compared to a method in which a Hounsfield unit (HU) threshold was used to detect hypoperfusion. Receiver operating characteristic area under the curve (AUC) and Matthew correlation coefficient (MCC) were calculated with their 95% confidence interval (CI).

**Results:**

The predicted segmentations of CNN showed AUC 0.87 (95% CI 0.82–0.91), those of HU-threshold method 0.79 (95% CI 0.74–0.84). The optimal global threshold values were CNN output probability ≥ 0.37 and ≤ -850 HU. Using these values, MCC was 0.46 (95% CI 0.29–0.59) for CNN and 0.35 (95% CI 0.18–0.48) for HU-threshold method (average difference in MCC in the bootstrap samples 0.11 (95% CI 0.05–0.16). A high CNN prediction probability was a strong predictor of CPE.

**Conclusions:**

We proposed a deep learning method for detecting hypoperfusion in CPE from CTPA. This model may help evaluating disease extent and supporting treatment planning.

## Key points

- The study aim was to detect hypoperfusion in chronic pulmonary embolism.
- Convolutional neural network was applied to computed tomography pulmonary angiography images.
- Good accuracy with moderate segmentation correlation to manual delineations was achieved.
- The model offered an improvement compared to a computed tomography density-based analysis.

## Background

Chronic thromboembolic pulmonary hypertension (CTEPH) is a late complication of pulmonary embolism [1, 2]. Unresolved thrombus is seen in 15–30% of patients 9–12 months after the initial pulmonary embolism [3, 4], which may organise and progress into chronic thromboembolic disease (CTED) [5]. Pulmonary vasculopathy may occur in the nonoccluded arteries [6]. Consequently, pulmonary vascular resistance and arterial pressure increase developing CTEPH [7] with a cumulative incidence of 0.1–9.1% within 2 years after a symptomatic pulmonary embolism event [8]. The incidence of CTED is estimated to be significantly higher [9]. Unlike other forms of pulmonary hypertension (PH), CTEPH is potentially curable surgically with pulmonary endarterectomy [10, 11]. If untreated, the median survival is less than 2 years if the mean pulmonary arterial pressure is > 30 mmHg [1, 2]. Hence, early diagnosis is cardinal for these patients.

The screening method of choice for CTEPH is ventilation/perfusion (V/Q) scan [12] while catheter-directed pulmonary angiography is considered the reference standard in confirming the diagnosis [13, 14]. Computed tomography pulmonary angiography (CTPA) can also be used to confirm the diagnosis of CTEPH [15–17] and estimate the severity of the pulmonary hypertension [18, 19]. Despite its low sensitivity in clot detection in the distal and subsegmental vessels [20], CTPA is fundamental in the diagnostic workup of CTEPH with a crucial role in assessing the feasibility of surgical treatment [17]. CTPA is noninvasive and more widely available than catheter-directed pulmonary angiography and has the advantage of evaluating the parenchymal and bronchial signs, the collateral circulation, and competing diagnoses [21].

In CTPA, the vascular signs for chronic pulmonary embolism (CPE) are complete occlusion of the pulmonary arteries or incomplete occlusion by nonobstructing bands, webs, or laminated thrombi [5, 21]. A typical parenchymal sign for CPE is mosaic perfusion, seen as well-demarcated regions of hypoattenuation representing hypoperfusion and hyperattenuating regions representing the blood flow to the patent pulmonary vascular bed [5, 21]. These radiological signs are frequently missed in the reports [22], and because of the disease insidious nature and the unspecific clinical presentation [23], the diagnosis is often delayed, with a median of 14 months after the initial symptoms [24]. A high index of suspicion is necessary for the diagnosis [21].

Several techniques have been developed for computer-aided detection and diagnosis of pulmonary embolism [25, 26]. However, no studies have been done on artificial intelligence techniques to detect parenchymal changes from CTPA relating to hypoperfusion in CPE. An automatic tool detecting these perfusion defects from CTPA images could potentially aid in diagnostics, evaluating the extent of the disease, and treatment planning of chronic pulmonary embolism. This study aimed to train a convolutional neural network (CNN) to detect the hypoperfused regions affected by CPE from CTPA images and evaluate the algorithm performance in this task.

## Methods

### Subjects and imaging parameters

We retrospectively reviewed reports of V/Q scans performed in Helsinki University hospital between January 2017 and December 2019. Based on the reports, 30 patients with findings suggestive of CPE in the V/Q scan were selected for the study. Also, 31 patients with no signs of pulmonary embolism in the V/Q scan were selected.

The inclusion criteria for the positive cases were a positive V/Q scan for CPE and a CTPA with signs of CPE performed in our hospital district within 3 months before or after the positive V/Q scan before treatment without signs of acute pulmonary embolism. Cases with no possibility to delineate areas of hypoperfusion at CTPA were excluded from the study. The negative patients’ inclusion criteria were a negative V/Q scan for acute or chronic pulmonary embolism and a negative CTPA for acute or chronic pulmonary embolism performed within three months of the negative V/Q scan. In both groups, CTPA studies, in which the radiological signs of a parenchymal disease unrelated to CPE (*e.g.*, hyperattenuation caused by talcosis) extended over two thirds of the lung volume, were excluded. Artefacts caused by foreign material that covered more than one third of the lung volume in the CTPA was a criterion for exclusion in both groups.

Five patients with positive findings were excluded from the study for the lack of presurgical CTPA in the picture archive, a lack of visible parenchymal hypoattenuation in the CTPA, lungs completely affected by CPE on the V/Q scan without demarcating difference of attenuation at CTPA, no CTED or CTEPH diagnosed clinically, and inclusion of a massive acute or chronic thromboembolism. Six patients with CPE-negative findings were excluded based on too large slice thickness on CTPA in our picture archive, parenchymal lesions covering over two thirds of the lung volume related to another disease (talcosis), acute pulmonary embolism, a suspicion of CPE at CTPA (two patients), and significant artefacts on CTPA related to prior surgery. After the exclusion, 25 positive (group A) and 25 negative (group B) cases were included in the study. Patient demographics are shown in Table 1.

**Table 1:** Patient demographics

	Median age (years, min–max)	Males	Females	CTEPH patients	CTED patients
Group A	67 (21–82)	8	17	18	7
Group B	67 (33–88)	11	14	0	0
All	67 (21–88)	19	31	18	7

*CTEPH* Chronic thromboembolic hypertension, *CTED* Chronic thromboembolic disease

In group A, 18 patients had a clinical diagnosis of CTEPH, clinically defined as the mean pulmonary arterial pressure > 25 mmHg at rest, pulmonary capillary wedge pressure < 15 mmHg documented with a right heart catheterisation, persisting mismatched perfusion defect in V/Q scan with specific diagnostic signs for CTEPH on CTPA or conventional pulmonary angiography [27]. Seven patients had CTED, defined with the same criteria as mentioned above, excluding pulmonary hypertension [28]. Seventeen patients with CTEPH had pulmonary hypertension confirmed with a right heart catheterisation. One CTEPH patient had only an echocardiogram done for the diagnosis. A normal pulmonary arterial pressure was confirmed with a right heart catheterisation for one of the patients with CTED. The rest did not warrant a right heart catheterisation.

The CTPA studies were performed before treatment on various scanners in multiple hospitals within the Hospital District of Helsinki and Uusimaa. The protocol was defined by the joint municipal authority for specialised healthcare. Different tube voltages were used depending on the scanner and the size of the patient (see Table 2 for details). All data were anonymised and stored on a server running the Extensible Neuroimaging Archive Toolkit version 1.1.6 [29].

**Table 2:** Number of patients imaged with each computed tomography scanner and tube voltage

	Tube voltage				
Scanner model (manufacturer)	80 kV	100 kV	120 kV	140 kV	Total
Somatom Definition AS (Siemens Healthineers)	0	2	1	0	**3**
Discovery HD (General Electric Healthcare)	0	4	0	0	**4**
Somatom Definition Edge (Siemens Healthineers)	1	6	1	2	**10**
Somatom Definition Flash (Siemens Healthineers)	0	9	2	0	**11**
Lightspeed VCT (General Electric Healthcare)	0	9	1	0	**10**
Aquilon Prime (Toshiba)	0	1	0	0	**1**
Revolution EVO (General Electric Healthcare)	0	2	2	0	**4**
Revolution HD (General Electric Healthcare)	1	5	0	1	**7**
**Total**	**2**	**38**	**7**	**3**	**50**

Patients were imaged with one of the eight scanner models depending on the referring hospital. Tube voltages ranged from 80 kV to 140 kV

### Preprocessing

A radiologist with over 5 years of experience manually segmented the hypoperfused regions on the CTPA images for the 25 patients in group A, using V/Q scan images as a reference for segmentation. To appreciate the minor differences in the parenchymal attenuation, grey-level mapping with a window width ranging from 200 to 900 and a window level ranging from -1,100 to -800 was used. Only the regions of hypoperfusion visible in the CTPA were segmented. Regions where hypoattenuation was suspectedly caused by lung disease other than CPE were not segmented. Areas with relevant artefacts relating to, *e.g.*, motion or beam hardening, were left unsegmented. Regions smaller than 5 mL were left unsegmented. Hyperattenuating lesions relating to the CPE were included in the segmentation, *e.g.*, lung infarctions and atelectasis in the diseased area if seen in the hypoperfused region. Segmentation was done on the axial images using the image processing and visualisation platform 3D Slicer [30].

Due to a large number of axial images, with a median of 335 (min–max, 111–570) per CTPA, we used a tool based on morphological contour interpolation for assistance in segmentation [31], which allowed any number of slices to be skipped between slices segmented manually. This tool interpolates the manually segmented slices by detecting and aligning overlapping shapes in the adjacent segmented slices and generating a transition sequence of one-pixel dilatation between the overlapping shapes, and a median of this sequence is taken as a result to fill the skipped slices [31, 32]. The radiologist segmented the hypoperfused regions to every 3–10 axial images by hand, and the complete segmentation was created by the interpolation tool. Contours of the interpolation were evaluated and approved by the radiologist before committing to the segmentation. If the radiologist disagreed with the interpolation, the contours of the interpolated segmentation were modified and corrected manually. In group A, segmentation was done in this manner to every axial CTPA image for the CNN training and analysis. In group B, there was no manual segmentation done for hypoperfusion. Additionally, in both groups, the lungs were segmented as a whole, and the CTPA image data outside of the lungs was excluded from the analysis.

We applied a 48%–12%–40% training-validation-test split, *i.e.*, 12 CTEPH positive and 12 negative CTPA volumes for training, 3 positives and 3 negatives for validation, 10 positives, and 10 negatives for testing. We split the data temporally: all the training and validation data had been imaged before all the test data. The hospital ethics committee approved the study. Informed consent was waived because of the retrospective design and anonymous clinical data used for the analysis.

### Neural network training

**Fig. 1:**
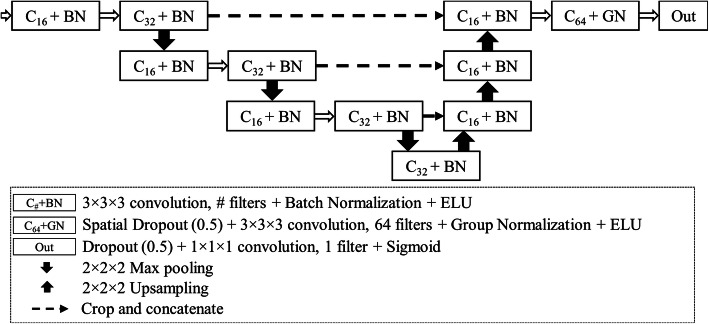
The convolutional neural network architecture was based on U-net with three max-pooling steps in the downsampling pathway and linear upsampling in the resolution recovery path. Convolutional layers (C) were followed by batch or group normalisation and exponential linear unit (ELU) activations. Skip connections with cropping were used between the matching resolution levels (dashed arrows). *C* Convolutional layer, *BN* Batch normalisation, *ELU* Exponential linear unit, *GN* Group normalisation

We trained a 12 layers deep U-net-type [33] CNN with three-dimensional convolutional layers (3 × 3 × 3 kernel size; valid padding; 16, 32, or 64 filters each), three max-pooling/upsampling steps with skip connections, and a single output neuron with sigmoid activation (see details in Fig. 1). Exponential linear units were used as activations in the hidden layers. The CNN was implemented using Keras in Tensorflow framework version 2.0 [34]. The compute tomography (CT) angiography volumes were resampled to uniform 1 × 1 × 1 mm^3^ and normalised by linearly shifting and scaling the -900…-600 HU range into -1…1 range. The training data were divided into 32 × 32 × 32 voxel patches, and the patches completely outside the lung region were rejected. These samples with borders extended by 30 voxels on each side (due to a valid padding scheme in all the convolutional layers) were fed into the network with batch size 16. During training, Adam optimiser [35] with 2 × 10^−4^ learning rate and Dice loss [36] against manual labels were used. The final model was achieved after 21 epochs, at which point the validation accuracy stopped improving. Also, no further improvement on validation data was achieved by lowering the learning rate after the 21st epoch.

### Performance and statistical testing

We compared the CNN model to a method which used a simple cutoff Hounsfield units (HU) threshold: voxels inside the lungs with a HU value below a specific threshold value were labelled as hypoattenuation. Hereafter, we will refer to this global threshold approach as the “naïve HU-threshold method”, in contrast to an active expert-selected threshold for each patient or region. We calculated the voxel segmentation receiver operating characteristic curves for these two methods and compared the area under curve (AUC) values produced by the CNN model and the naïve HU-threshold method. Due to inherent class imbalance in the dataset (21% of lung voxels in the total dataset were labelled as hypoperfused), we chose the Matthews correlation coefficient (MCC) [37] as the segmentation performance metric. Secondly, in contrast to the more commonly used F_1_ metric, which emphasises the positive class, MCC remains agnostic of which of the binary classes is selected “positive”. Therefore, we avoid imposing hypoattenuating areas to be more important than the remaining tissues in the segmentation performance evaluation. Confidence intervals (CIs) for the reported performance metrics were calculated using a bootstrap method by resampling with replacement with 10^5^ repeats and 95% significance level. The optimal threshold for visual inspections was chosen by maximising the test set balanced accuracy. We evaluated the test cases visually to assess the clinical relevance of the false positive and negative segmentations. We further investigated if the CNN probability or the mean HU could indicate the presence of the CPE.

## Results

### Segmentation performance

**Fig. 2:**
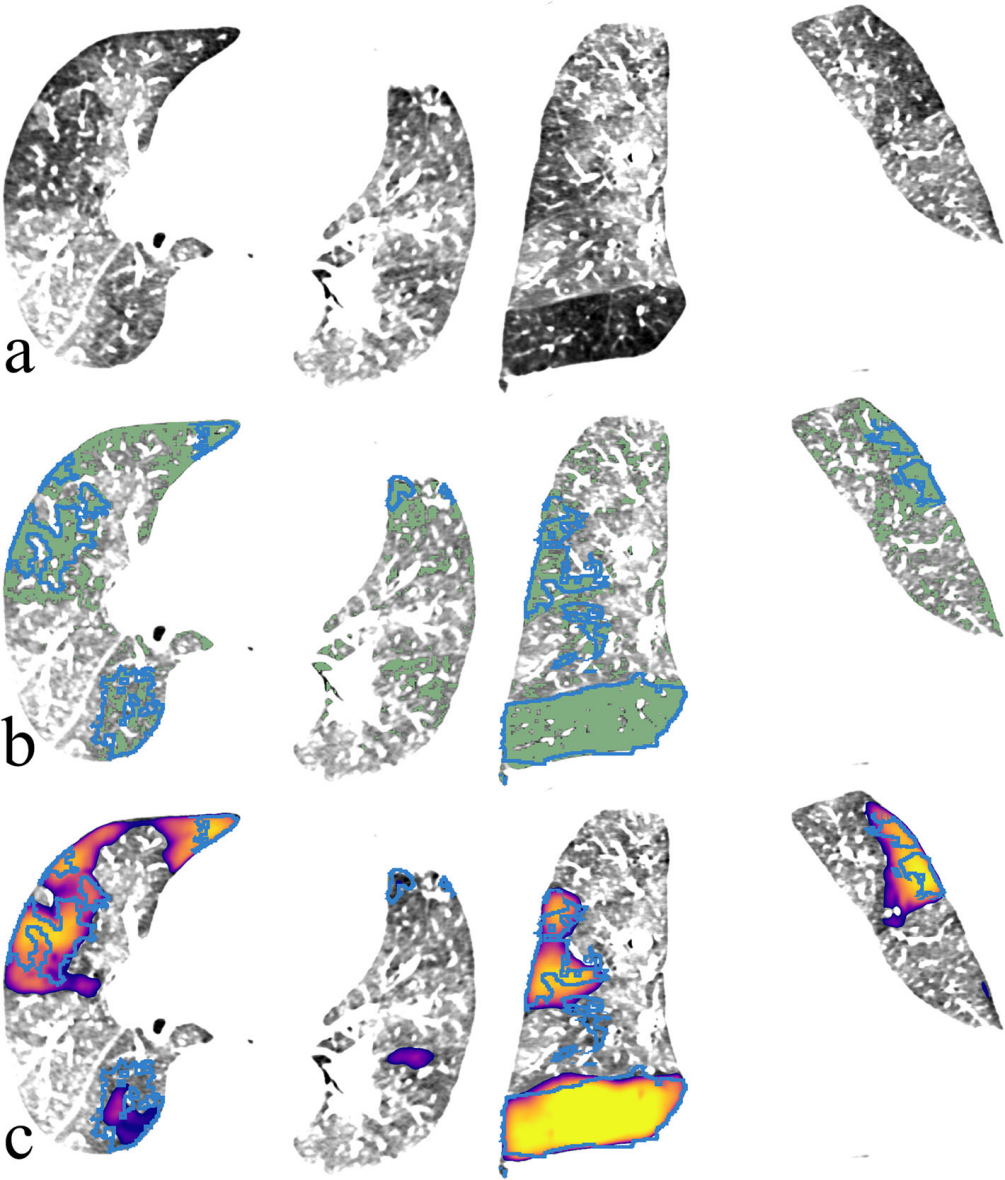
An example of computed tomography angiography volume (**a**) segmentations with good agreement to manual delineations (blue outline): HU threshold (≤ -850)-derived segmentation is shown in green (**b**), while the prediction of convolutional neural network is shown as colourmap (≥ 0.37 probability cutoff) (**c**). *HU* Hounsfield units

An example of manual, naïve HU-threshold and CNN segmentations is shown in Fig. 2. Time for manual segmentation was approximately 5 h per case.

**Fig 3:**
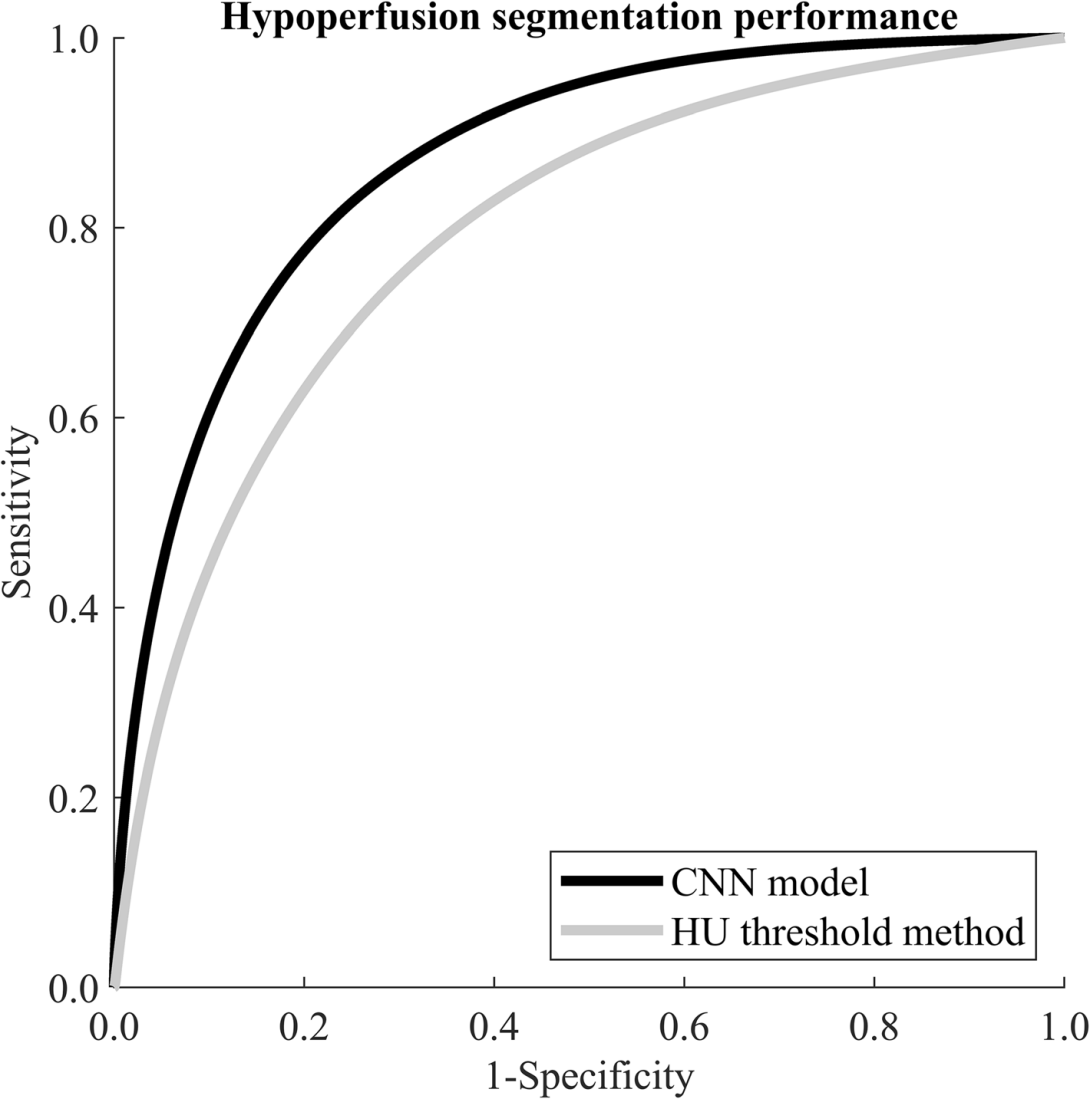
Receiver operating characteristic curves when applying the convolutional neural network (CNN) model or using a global naïve Hounsfield unit threshold method. The areas under the curves were 0.87 and 0.79, respectively. *HU* Hounsfield units

Using the naïve HU-threshold method over all the test samples, the ROC AUC was 0.79 (95% CI 0.74–0.84); using the CNN model the ROC AUC was 0.87 (0.82–0.91) (see Fig. 3). The optimal global threshold value was ≤ -850 HU for the threshold method and CNN output probability ≥ 0.37. We used these values in the following MCC and visual assessments. MCCs were 0.35 (95% CI 0.18–0.48) and 0.46 (95% CI 0.29–0.59), for the naïve HU-threshold method and the CNN model, respectively. The average difference in the MCCs between the two methods in the bootstrap samples was 0.11 (95% CI 0.05–0.16; positive value meaning CNN performed better). Because the 95% CI excluded zero, we conclude in favour of a statistically significant improvement achieved by using the CNN model.

### Visual assessment

**Fig. 4:**
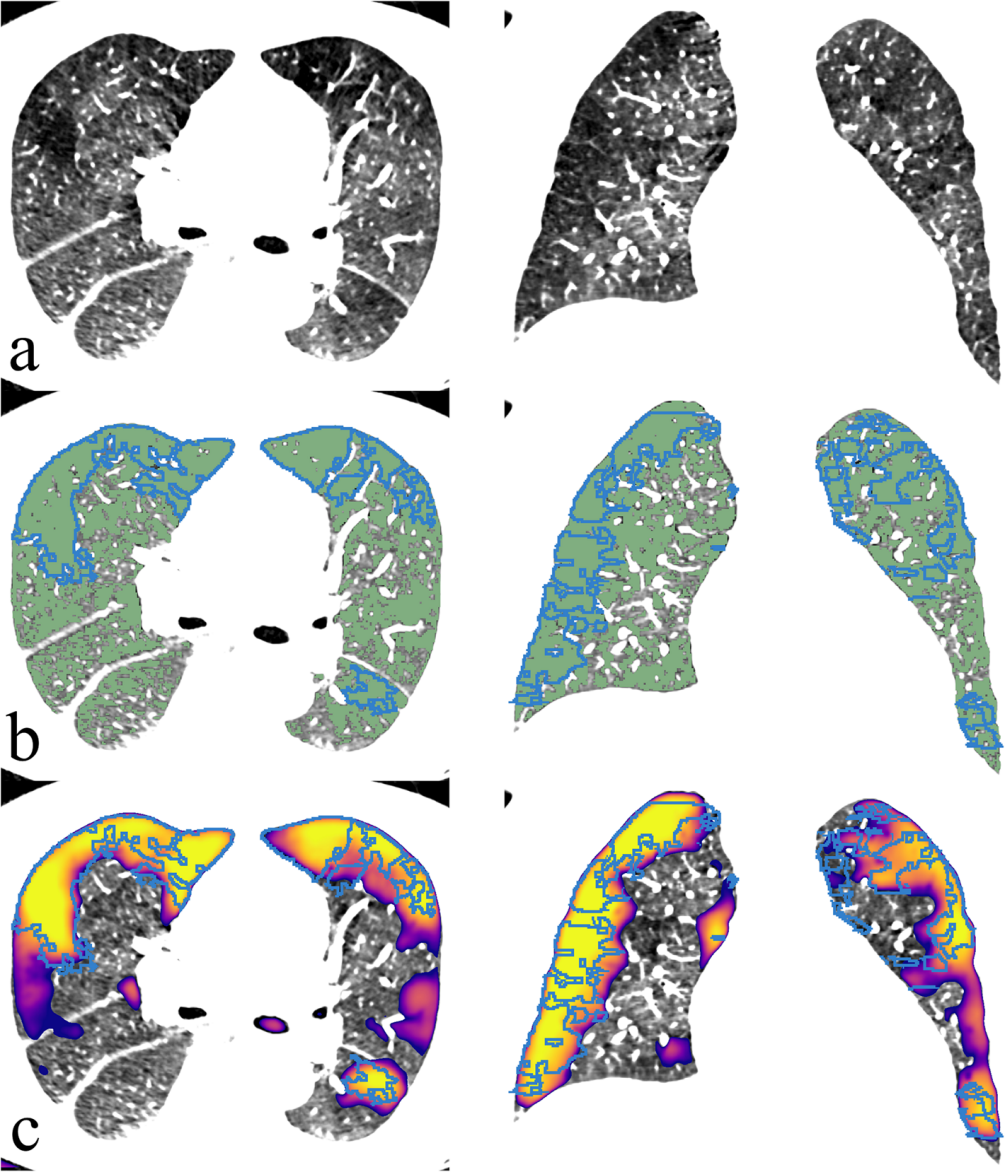
An example case (**a**) of an overall low attenuation where a predefined HU-threshold (≤ -850 HU shown in green in **b** performs suboptimally compared to the convolutional neural network model (**c**) when compared to manual segmentation (blue outlines). *HU* Hounsfield units

Complementary to voxel-wise performance evaluation, we reviewed each test set CTPA by visually assessing individual regions segmented by the CNN model and the naïve HU-threshold method (Fig. 4). Regions overlapping the manually segmented areas were defined as true positives. Regions not overlapping with the manually segmented areas were defined as false positives. Manually segmented regions not overlapping with the segmentation done by the prediction algorithm were defined as false negatives. If more than one part segmented by the prediction algorithm overlapped a same manually segmented region, they were all defined together as one true positive. We ignored prediction labels outside the lung parenchyma.

**Fig. 5:**
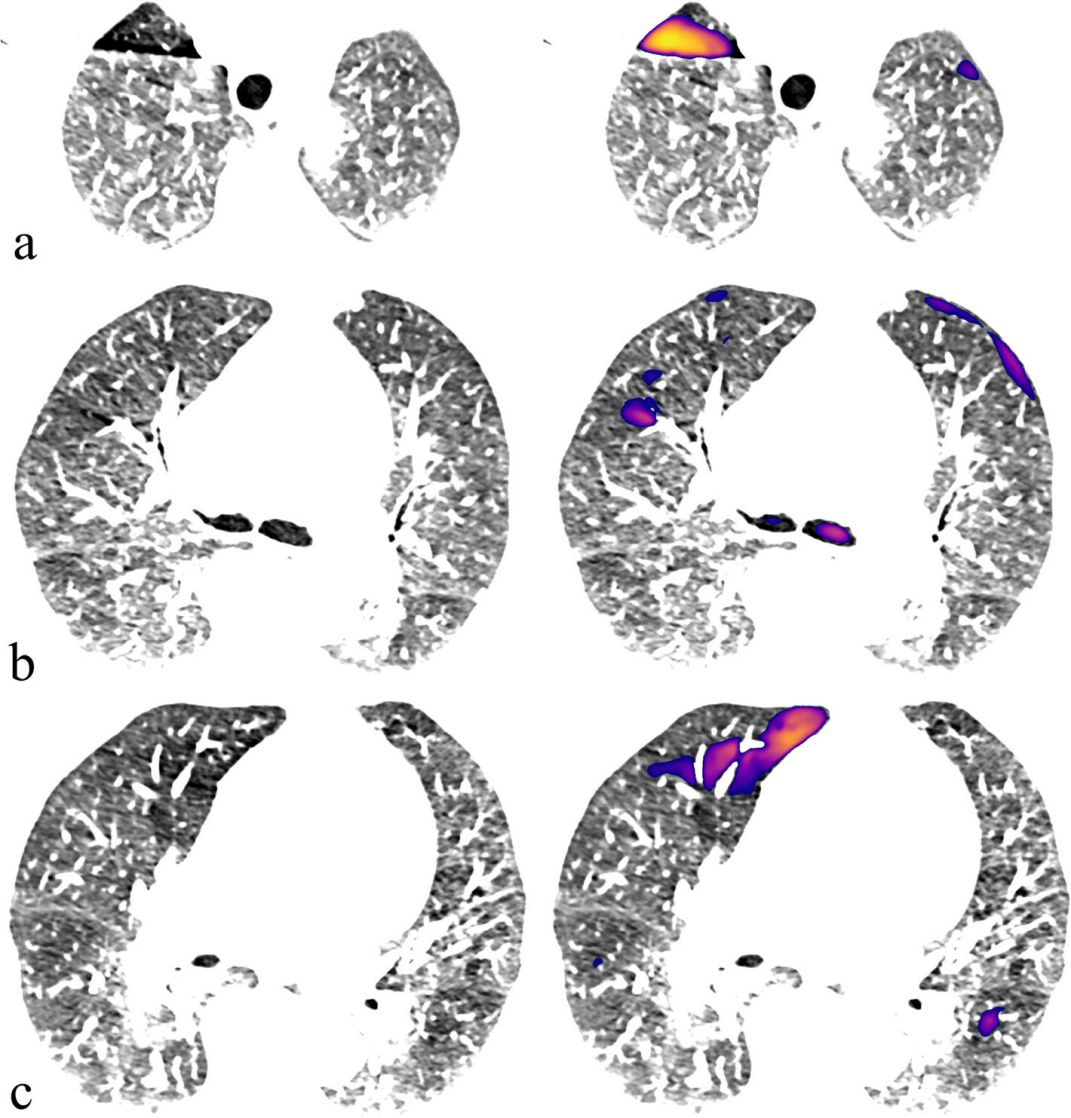
Common false negative convolutional neural network predictions were related to beam hardening artefacts seen adjacent to dense contrast material in the subclavian vein (**a**), around vessel (**b**) in subpleural sites (**b**), and adjacent to larger organs like the heart and the hilum (**c**)

In the CNN output, there were 63 independent false positive labels in group A and 111 false positive labels in group B, respectively. Beam hardening artefacts from the larger organs, including the heart, the hilum of the lung, the aorta, and the diaphragm, were seen in 17% of the false positives. In 16%, there were beam hardening artefacts relating to the contrast material bolus in either superior vena cava, subclavian, or brachiocephalic veins. Thirty-three per cent of the false positives were adjacent to smaller blood vessels and 15% were minor subpleural regions most likely related to beam hardening (Fig. 5).

**Fig. 6:**
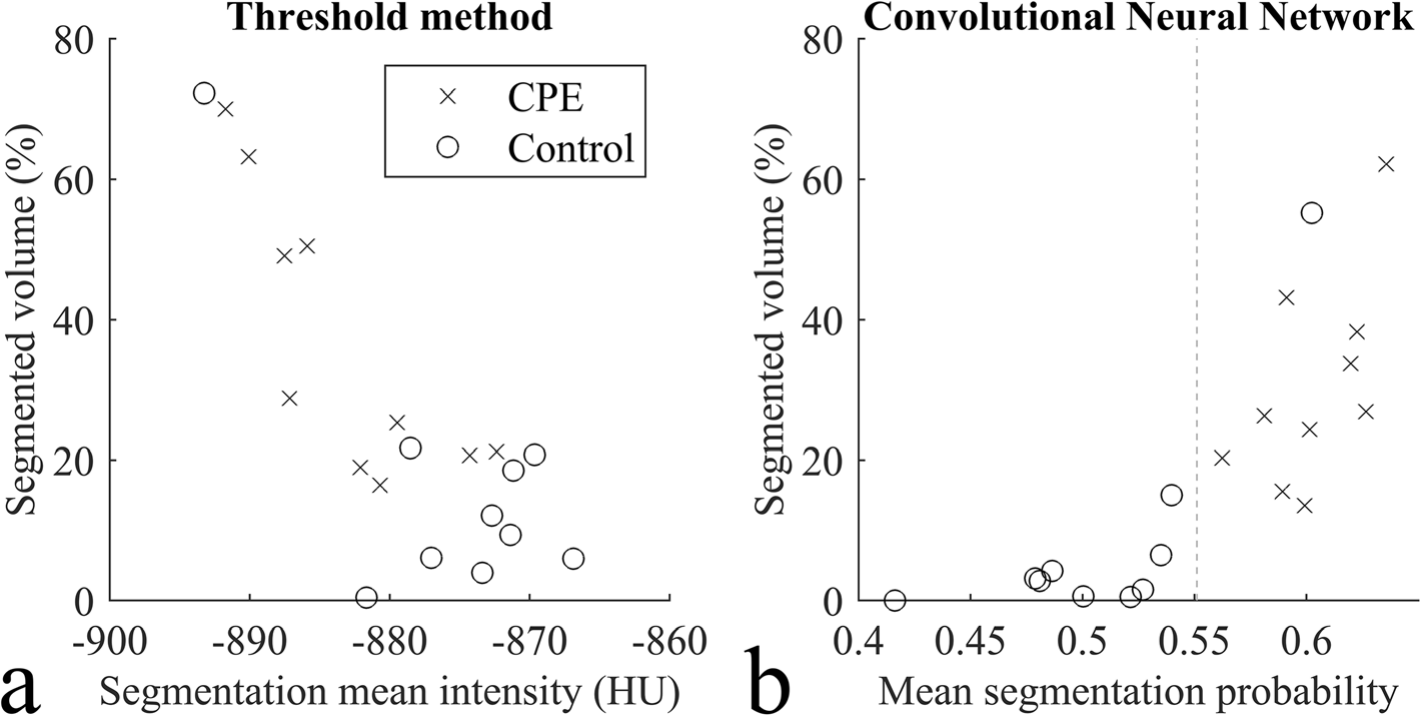
CPE cases (test set cases of group A) are indicated with crosses and the controls (test set cases of group B) with circles. *CPE* Chronic pulmonary embolism, *HU* Hounsfield units

**Fig. 7:**
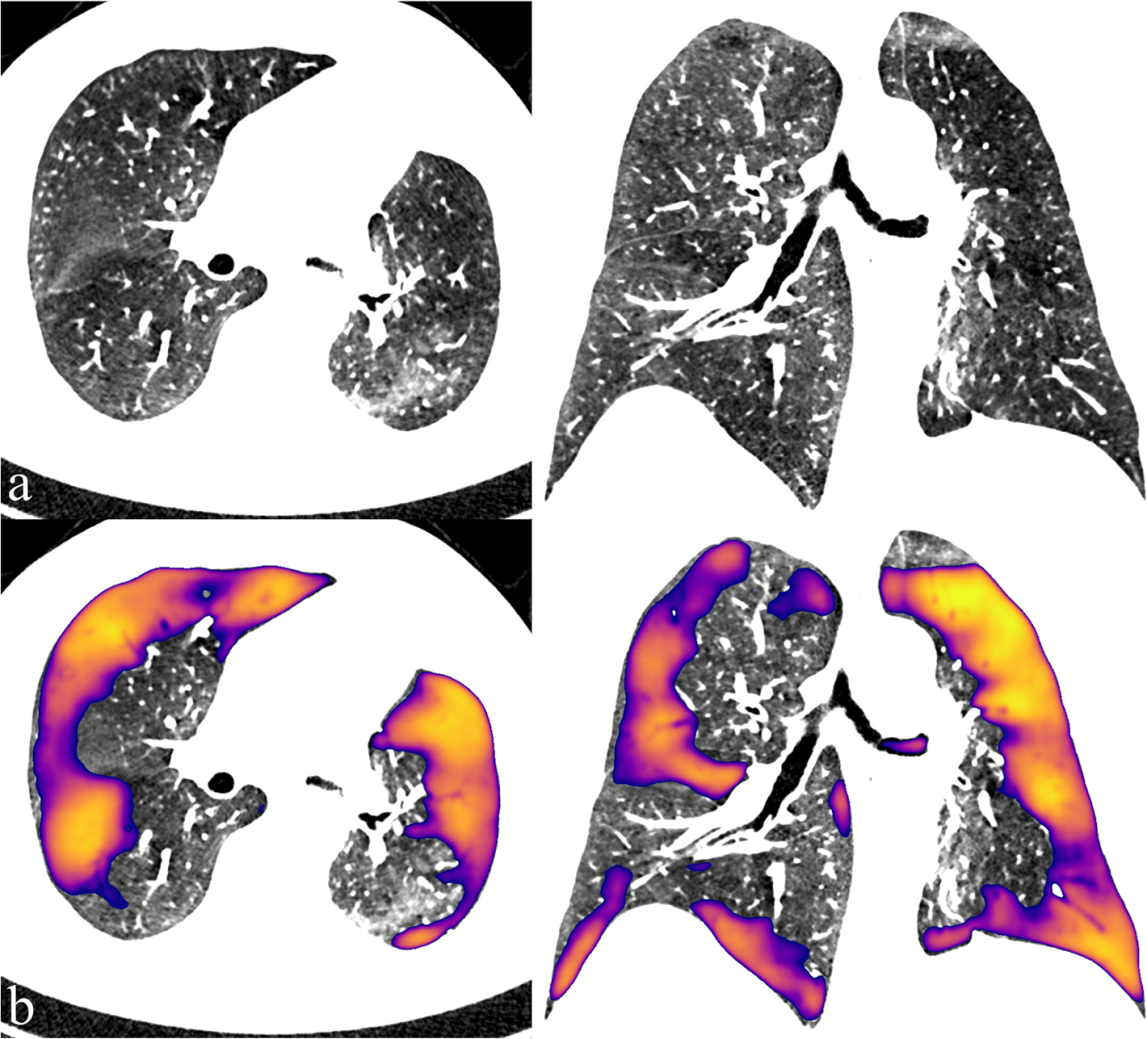
One patient with previous upper lobe left pneumonectomy, and the remaining lung parenchyma was low in HU, resulting in large false positive predictions by the convolutional neural network. *HU* Hounsfield units

Twelve areas of varying sizes were labelled positive by the CNN, which were incorrectly labelled negative in the preprocessing. Nine per cent of the false positives remained unspecific by nature. In one case, there was air trapping relating to a mucus plug in a bronchus. One patient in group B had gone through left upper lobe pneumonectomy in the past, and the majority of the remaining lung volume was detected as false positive by the CNN algorithm and HU-threshold method (false positive outlier, Fig. 6b; Fig. 7).

Of the 137 manually segmented lesions, 133 were correctly labelled positive by the CNN. There were both overestimation and underestimation of these lesions. The overestimation of 8 true positive labels was related to hypoattenuation caused by beam hardening artefacts. Otherwise, the cause of the overestimation and underestimation remained unspecific.

In the naïve HU threshold algorithm, each voxel below the certain threshold was labelled as positive. With the optimal HU-threshold of < -850, the result consisted of numerous voxel clouds of different sizes, including single voxels, because no voxel clustering was used. As this method labelled a myriad of regions positive, ranging from one voxel size to larger areas, it was not reasonable to count them. Hence, we present the visual assessment of the naïve HU-threshold method descriptively.

The naïve HU threshold algorithm responded to general noise in the lung parenchyma consisting of both morphologic variegation and imaging noise, which can be seen in the healthy and disease-associated lung parenchyma. Hypoattenuating voxels caused by artefacts were also marked as false positives. Each manually segmented hypoperfusion area contained at least one voxel labelled as true positive. As shown in Fig. 6, both the mean over the lung tissue with < -850 HU and the average CNN prediction probability, *i.e.*, network’s confidence in the voxel segmentation, indicate the presence of CPE. Using the CNN and value 0.55 (dashed line in Fig. 6b), all but one test case was correctly categorised. The network inference time of the CNN model for a single CTPA in the test set was between 17 and 32 s using an NVIDIA Quadro M6000 24GB graphics card.

## Discussion

As far as we know, this is the first study to evaluate the feasibility of three-dimensional CNN in the detection of lung hypoperfusion from CTPA images in CPE. Our CNN algorithm showed an encouraging performance in detecting these lesions on a separate testing set. Since there are no previous studies to compare our results to, we compared the CNN algorithm to a method based on a simple HU cutoff threshold. Using even the HU threshold segmentation could help visual evaluation, but according to our results, the CNN algorithm could offer a significant improvement with a 0.87 ROC-AUC and 0.46 MCC compared to the 0.79 and 0.35 for the naïve HU-threshold method, respectively.

The average CNN prediction probability indicates the presence of CPE, and all but one of the CPE and control cases in the test set were correctly categorised using a probability value 0.55. Therefore, we propose that in addition to the lesion segmentation, the disease overall presence could be inferred using a CNN-based approach.

The CNN algorithm recognised most (97%) of the manually segmented lesions. Majority (82%) of the false positive CNN labels were small and related to beam hardening artefacts from dense organs, which seemed to be the most challenging for the CNN to differentiate from true positive lesions. However, one patient had extensive false positive labels covering most of the lung volume. This patient had undergone left upper lobe pneumonectomy and mean HU of the lung parenchyma was one of the lowest in the study group. After lobectomy, the remaining lung has a relative increase of air and a decrease of the lung parenchyma in the thorax seen as hyperlucency on a CT image [38]. This is likely the reason for the hyperlucency in this patient’s lung and the CNN false positive labelling.

There was both overestimation and underestimation in the sizes of the hypoperfused lesions. The overestimation was partly related to the beam hardening artefacts mentioned earlier, but in most cases, the overestimation and underestimation remained unspecific. Since accurate delineation of the hypoattenuating regions in the CTPA was challenging, we had to compromise the manual segmentation in cases where the borders between affected and unaffected areas were gradient. This might partially explain the overestimation and underestimation of the lesion sizes in the labels output by the CNN algorithm.

CNNs have been previously applied in pulmonary embolism detection based on labelling the occluding clots seen in CTPA as filling defects of intravascular contrast material in pulmonary arteries [39–42]. These studies have focused mainly on acute pulmonary embolism. We took a different approach, and instead of the actual vascular defects, we focused on the hypoattenuation in the lung parenchyma relating to the hypoperfusion caused by chronic pulmonary embolism. Öman et al. (2019) have successfully implemented this type of approach to detect acute stroke lesions from CT angiography images with a CNN based algorithm [43]. However, since the lungs are composed largely of air, the differences in parenchymal enhancement are subtler between the hypo- and hyperperfused regions at CTPA than at CT angiography of the brain. Another significant difference in the setting is that a single thrombus usually causes the ischemic stroke, whereas chronic pulmonary embolism affects many branches of the pulmonary vasculature by various degrees of obstruction. Unlike the brain, the attenuation of the lung is sensitive to regional abnormalities of perfusion or aeration, which can be seen as heterogeneous lung attenuation on CT [44].

In patients with CPE, the areas of low attenuation on CTPA represent areas of hypoperfusion [45], which is due to both the obstructing vascular impairment caused by chronic thrombus and the vasculopathy in the regions clear of the thrombus [6]. The low attenuation may also be due to air trapping in CTEPH patients relating to abnormalities in the affected regions' small airways [46]. Our study had only CTPA studies without expiration images, so we could not definitely differentiate the hypoperfusion and air trapping. However, regardless of the cause, hypoattenuation is associated with the regions affected by CTE, and, *i.e.*, Bartalena et al. [47] showed that the hypoattenuating regions seen in CTPA correlated well with hypoperfusion seen in perfusion scintigraphy of patients with pulmonary hypertension including CTEPH.

Absence of mosaic perfusion is not exclusive for CPE as in some studies up to 26–45% of patients with CTEPH did not present mosaic perfusion at CTPA [12, 48]. The mosaic perfusion is also not specific for CPE, and various diseases mimic this pattern [49]. Hence, this type of CNN algorithm would not be a comprehensive tool for the diagnosis or exclusion of CPE, but it may help assess the extent of the disease and treatment planning. With a processing time well below 1 min, the CNN model could be implemented as a low-latency clinical application in the picture archiving and communication system by overlaying the prediction on top of the images or by reporting the volume(s) of the hypoperfused regions. This type of application might also assist the radiologist in diagnosing CPE, which is often misdiagnosed and difficult to detect, especially if combined with an algorithm assessing the vascular defects. A more extensive database is needed to conclude the feasibility of CNN algorithms in a clinical setting and with automatic methods of detecting the vascular defects.

A limitation of our study was that the labels were manually segmented by a single radiologist and contained an unknown interobserver variability and individual bias level. Contributing to this label noise was the lack of expiration CT scans, and definite differentiation between hypoperfusion and air trapping was not possible. Additionally, borders between the regions with differing perfusion were gradient in many cases, and exact demarcation was not always univocal during the pre-processing, which might explain some of the overestimation and underestimation of the lesions by the CNN algorithm.

The patients in the study had different parenchymal changes relating to other diseases (*e.g.*, scars, atelectasis, pleural fluid, emphysema, inflammatory lesions), and all the patients who had these types of findings extended to over two thirds of the lung volume were excluded from the study. Although this exclusion criterion presents an unknown amount of bias in the sample profile, we found it necessary. Otherwise, the manual segmentation could not have been done objectively, leaving the segmentations too imprecise. To avoid inaccurate labelling, the smallest regions of hypoperfusion and regions with undefined borders relating to noise and artefacts were left manually unlabelled. The segmentation was done correspondingly for the whole group A (divided for the training, validation and the test sets). This had an unknown effect on the results as the CNN labelled some of these small regions correctly as hypoperfusion. Still, they presented as false positives since the manual segmentation was considered the ground truth. New practical methods for improved manual segmentation and labelling the parenchymal lesions in the lungs need to be explored. One such tool is the morphological contour interpolation introduced by Zukić et al. [31], which implements a morphology-based interslice interpolation proposed by Albu et al. [32]. In our study, this tool turned out invaluable for the manual segmentation since the lesions were large in number and volume with irregular shapes and borders, and not always well demarcated in every CT slice.

Finally, we acknowledge as a study limitation the small sample size, for which only a modest anatomical and pathological variation were available during training. Also, the small number of validation cases may have led to a suboptimal stopping point and model selection. Nevertheless, to address the resulting problem of unknown generalizability, the data portion dedicated to testing was kept relatively high in the study design. Likely, a big data approach (*e.g.*, training with additional, more readily available, non-CPE cases) would be beneficial, especially in decreasing the extent and frequency of false-positive regions.

In conclusion, this study demonstrated the feasibility of a deep learning algorithm for the detection of hypoperfusion in CPE from CTPA with a good performance. These encouraging findings suggest that CNNs could be used as an automated method for assisting the clinician and radiologist in diagnosis and treatment planning for patients with CPE.

## Data Availability

The trained deep learning models are available on reasonable request sent to the corresponding author.

## Abbreviations

AUC: Area under curve
CI: Confidence interval
CPE: Chronic pulmonary embolism
CT: Computed tomography
CTED: Chronic thromboembolic disease
CTEPH: Chronic thromboembolic pulmonary hypertension
CTPA: CT pulmonary angiography
MCC: Matthews correlation coefficient
V/Q: Ventilation/perfusion
